# Elevated serum TSH concentrations are associated with higher BMI Z-scores in southern Iranian children and adolescents

**DOI:** 10.1186/s13044-020-00084-9

**Published:** 2020-06-13

**Authors:** Ashkan Habib, Mohadeseh Molayemat, Asadollah Habib

**Affiliations:** 1 School of Medicine, Shiraz University of Medical Sciences, Shiraz, Iran; 2 Department of Endocrinology, School of Medicine, Kazerun Branch, Islamic Azad University., First Floor, Zafar Building, Zand St, PO Box: 71384-37984, Shiraz, Iran

**Keywords:** BMI, BMI Z-score, Children, Thyroid, Hypothyroidism, Iran

## Abstract

**Background:**

Subclinical hypothyroidism is defined as elevated TSH levels while T4 or FT4 levels are normal. Elevated TSH levels are linked with obesity in adults. In a recent meta-analysis in Iran, 6.1% of children below 18 had obesity. Due to the low number of studies on the subject in children we, designed the study to assess the relation between BMI Z-score and TSH levels in children and adolescence.

**Method:**

This cross-sectional study was performed in a pediatric endocrinology clinic in Shiraz. Children aged between 2 to 18 years that came to the clinic for routine growth assessment follow up from January till April 2018 were considered. 850 children including 365 boys and 485 girls were included.

**Results:**

Prevalence of subclinical hypothyroidism is increased in higher BMI groups. 9.9, 13.8, 17.2 and 20.5% of underweight, healthy weight, overweight and obese had subclinical hypothyroidism respectively. Obese and overweight participants had higher odds of subclinical hypothyroidism than those who were not (OR:1.649, *P* = 0.010, CI95% 1.126–2.413). On the other hand, Subclinical hypothyroid participants had higher odds of overweight or obesity than those who were euthyroid (OR:1.650, P = 0.010, CI95% 1.128–2.413). When TSH is set as a dependent value, TSH level is increased (β = 0.126, r = 0.125, *P* = 0.001) with an increase in BMI Z-score. When BMI Z-score is set as a dependent value, BMI Z-score is increased (β = 0.113, r = 0.243, P = 0.001) with an increase in TSH level.

**Conclusion:**

BMI Z-score and elevated TSH levels are positively correlated however studies should be performed on establishing the causality.

## Introduction

Subclinical hypothyroidism is defined as elevated TSH levels while T4 or FT4 levels are normal [1]. It is a common disorder with a prevalence of 1 to 10% in Adult Community, [2, 3] while in the pediatrics population subclinical hypothyroidism is slightly lower than 2% [2, 4]. In adults, subclinical hypothyroidism is linked with abnormal lipid profiles, early signs of impaired cognitive function and increased risk of progression into overt hypothyroidism [2, 3, 5–8]. Elevated TSH levels are linked with obesity [9], and they are found to be reversible after weight loss, whether being attained through bariatric surgery or diet [10–15]. These changes are also found to be a consequence of obesity not the cause of it [12, 13, 16]. Similar mechanisms have been hypothesized to explain this elevated TSH levels in the obese population, including an adaptation to increased resting energy expenditure [10, 16], increased production of leptin-mediated Pro TRH [17–19], increased number of T3 receptors in the hypothalamus [20] and variations in peripheral deiodinase activity [19, 20].

Meanwhile, obesity is currently the most common metabolic disorder in many countries [21–23]. The World Health Organization (WHO) categorizes childhood obesity as one of the most serious global health challenges of the twenty-first century affecting many low and middle-income countries [24]. Studies have also shown that it is very likely that childhood obesity persists into adulthood [25]. In a recent meta-analysis in Iran, 6.1% of children below 18 had obesity. The study also showed a descending trend of obesity prevalence in Iran [26]. Due to the low number of studies correlating TSH and BMI in children we hypothesized that this relation also exists in patients under the age of 18 and obese children have a higher chance of subclinical hypothyroidism. As a result, we designed the study to assess the correlation between BMI Z-score and TSH levels in children and adolescence.

## Method

This is a cross-sectional study performed on data from children with an age of 2 to 18 years that came to a pediatric endocrinology growth assessment clinic for routine growth follow up from January till April 2018 located in the city of Shiraz. The clinic is open to patients 6 days a week with morning and evening shifts. After consent from parents, children were checked for serum thyroid profile levels simultaneously in a non-fast state between 8 AM and 6 PM in a single laboratory. Inclusion criteria for this study were: 1- an age of 2-18 years; 2- presence of normal free T4 (0. 8-1.8 ng/dL); 3- TSH between 0.3 and < 10 mIU/L. Exclusion criteria were 1- Those children who were on levothyroxine therapy at the time of assessment; 2- Ongoing use of medications that may interfere with thyroid function test as anti-thyroid medications, corticosteroids, oral contraceptives, thiazides.

850 children including 365 (42.9%) boys and 485 (57.1%) girls had full inclusion criteria and were selected for the study. Children’s weight were measured lightly dressed and without shoes using Seca scale with a precision of 0.1 kg while Height was measured to the nearest 0.1 cm using a stadiometer. UptoDate calculators (based on CDC growth charts) were used for measurement of BMI, SDS BMI, BMI percentile. BMI Z-score was calculated by the LMS (lambda, mu, sigma) method based on the reference of BMI distribution of CDC growth charts.

### BMI-SDS = ((BMI/m)L − 1)/LS

The study group was separated into 2–9 year old and 10-18 year old age groups representing before and after start of puberty. Subjects below 5th percentile (<− 1.65 BMI Z-score) were categorized as underweight, between 5th and 85th percentile (− 1.65 − + 1.04 BMI Z-score) as healthy weight, between 85th and 95th (+ 1.04 − + 1.65 BMI Z-score) as overweight and above 95th percentile (> + 1.65 BMI Z-score) as obese.

Serum TSH was measured using Cobas e411 Analyzer (Mannheim, Germany) with electrochemiluminescence immunoassay (ECLIA) method. Assay performance was controlled using Elecsys PreciControl Universal. Auto Analyser was calibrated using Elecsys TSH CalSet. Inter-assay coefficients of variation (CVs) for TSH are 1.56% for 1.37 mIU/L and 0.08% for 8.62 mIU/L, respectively. The study was approved by the Islamic Azad University, Kazerun Branch institutional review committee. (reference 1398.125).

TSH levels equal or above 5 were considered abnormal. All participants with high TSH levels were considered for a second remeasurement. For these participants, second TSH levels were considered for the study.

Participants with TSH levels equal or above 5 IU/m and lower than 10 IU/ml with normal free T4 levels were categorized as subclinical hypothyroid children based on the 2014 European Thyroid Association guideline on management of subclinical hypothyroidism in children [27]. TSH levels above 10 IU/ml are considered overt hypothyroidism and as a result were not included in this study.

### Statistical analysis

Comparisons were performed by using ANOVA for continuous variables in Tables 1 and 2 and the Student t-test for Table 3. The relation between BMI Z-score and TSH level was evaluated using multiple variable linear regression adjusted for age and gender. For categorical variables in Tables 4 and 5, comparisons were performed by using chi_squared test. Odds ratios for subclinical hypothyroidism in overweight and obese subjects and likewise, overweight and obesity in subclinical hypothyroid subjects was calculated using logistic regression, adjusted for age and gender. A value of *p* < 0.05 was considered statistically significant in all comparisons with a confidence interval of 95%. All statistical analysis was performed by using SPSS software version 25.0 (SPSS, Chicago, IL, USA).

**Table 1:** Anthropometric and laboratory values of the study subjects

Based on BMI category						Based on thyroid status		
	Underweight (141)	Healthy weight (369)	Overweight (116)	Obese (224)	P	Euthyroid (719)	Subclinical Hypothyroid (131)	P
Age	8.80 ± 3.48	9.57 ± 3.56	11.03 ± 3.30	10.38 ± 2.94	< 0.001*	9.84 ± 3.45	9.98 ± 3.26	0.663
Height	123.78 ± 19.03	131.08 ± 20.63	142.44 ± 16.40	142.72 ± 15.01	< 0.001*	133.95 ± 20.10	137.43 ± 17.52	0.064
Weight	20.97 ± 7.67	30.54 ± 13.00	46.54 ± 15.83	57.72 ± 19.90	< 0.001*	37.72 ± 20.25	41.49 ± 19.31	0.049*
Male (%)	56.0%	36.0%	36.2%	49.6%	< 0.001*	42.6%	45.0%	0.598
BMI	13.23 ± 1.07	16.84 ± 2.47	22.12 ± 2.91	27.49 ± 5.21	< 0.001*	19.57 ± 6.22	20.88 ± 6.01	0.026*
BMI Z-score	−2.86 ± 1.13	−0.25 ± 0.78	1.36 ± 0.18	2.18 ± 0.39	< 0.001*	0.10 ± 1.8334	0.60 ± 1.81	0.005*
TSH	2.89 ± 1.64	3.05 ± 1.85	3.22 ± 1.77	3.53 ± 2.13	0.005*	2.54 ± 1.14	6.65 ± 1.41	< 0.001*
FT4	1.41 ± 0.24	1.43 ± 0.25	1.39 ± 0.27	1.39 ± 0.26	0.221	1.41 ± 0.25	1.43 ± 0.27	0.247

**Abbreviations:** BMI, body mass index; TSH, thyroid stimulating hormone; FT4, free T4

**Table 2:** Mean thyroid stimulating hormone and free T4 levels based on subject BMI group

			Underweight	Healthy weight	Overweight	Obese	P
All ages	All (850)	TSH	2.89 ± 1.64	3.05 ± 1.85	3.22 ± 1.77	3.53 ± 2.13	0.005*
		FT4	1.41 ± 0.24	1.43 ± 0.25	1.39 ± 0.27	1.39 ± 0.26	0.221
	Male (365)	TSH	2.78 ± 1.55	3.16 ± 1.77	3.145 ± 1.69	3.75 ± 1.94	0.002*
		FT4	1.41 ± 0.23	1.41 ± 0.24	1.30 ± 0.26	1.42 ± 0.23	0.040*
	Female (485)	TSH	3.02 ± 1.75	3.00 ± 1.90	3.27 ± 1.83	3.31 ± 2.28	0.457
		FT4	1.40 ± 0.25	1.44 ± 0.26	1.44 ± 0.26	1.36 ± 0.28	0.045*
2–9 year olds	All (429)	TSH	2.79 ± 1.58	2.98 ± 1.62	3.24 ± 1.83	3.64 ± 2.18	0.005*
		FT4	1.44 ± 0.25	1.45 ± 0.24	1.44 ± 0.25	1.40 ± 0.27	0.450
	Male (155)	TSH	2.71 ± 1.66	3.34 ± 1.84	2.23 ± 1.37	4.05 ± 1.82	0.003*
		FT4	1.45 ± 0.24	1.47 ± 0.21	1.44 ± 0.19	1.43 ± 0.23	0.872
	Female (274)	TSH	2.88 ± 1.50	2.81 ± 1.48	3.43 ± 1.86	3.37 ± 2.36	0.084
		FT4	1.42 ± 0.25	1.44 ± 0.25	1.44 ± 0.26	1.38 ± 0.29	0.504
10-18 year olds	All (421)	TSH	3.03 ± 1.72	3.15 ± 2.10	3.21 ± 1.74	3.44 ± 2.09	0.521
		FT4	1.36 ± 0.23	1.41 ± 0.27	1.36 ± 0.27	1.38 ± 0.25	0.463
	Male (210)	TSH	2.88 ± 1.41	2.99 ± 1.70	3.33 ± 1.70	3.58 ± 2.00	0.138
		FT4	1.36 ± 0.21	1.36 ± 0.26	1.27 ± 0.26	1.41 ± 0.23	0.057
	Female (211)	TSH	3.24 ± 2.11	3.26 ± 2.34	3.09 ± 1.80	3.24 ± 2.20	0.984
		FT4	1.36 ± 0.25	1.44 ± 0.27	1.44 ± 0.26	1.34 ± 0.27	0.087

**Abbreviations:** BMI, body mass index; TSH, thyroid stimulating hormone; FT4, free T4

**Table 3:** Mean body mass index (BMI) and BMI Z-score based on subject thyroid status

			Euthyroid	Subclinical Hypothyroid	P
All ages	All (850)	BMI	19.57 ± 6.22	20.88 ± 6.01	0.026*
		BMI Z-score	0.10 ± 1.8334	0.60 ± 1.81	0.005*
	Male (365)	BMI	19.73 ± 6.57	21.57 ± 6.73	0.051
		BMI Z-score	−0.05 ± 2.04	0.51 ± 2.12	0.054
	Female (485)	BMI	19.44 ± 5.94	20.31 ± 5.34	0.247
		BMI Z-score	0.22 ± 1.66	0.67 ± 1.52	0.032*
2-9 year olds	All (429)	BMI	17.30 ± 4.60	19.57 ± 5.71	0.003*
		BMI Z-score	−0.15 ± 1.88	0.59 ± 1.99	0.004*
	Male (155)	BMI	16.61 ± 4.50	18.51 ± 5.84	0.120
		BMI Z-score	−0.59 ± 2.15	−0.05 ± 2.55	0.248
	Female (274)	BMI	17.68 ± 4.62	20.27 ± 5.58	0.001*
		BMI Z-score	0.10 ± 1.67	1.00 ± 1.39	0.000*
10-18 year olds	All (421)	BMI	21.85 ± 6.78	22.29 ± 6.06	0.635
		BMI Z-score	0.36 ± 1.75	0.61 ± 1.61	0.288
	Male (210)	BMI	21.98 ± 6.91	24.16 ± 6.40	0.099
		BMI Z-score	0.34 ± 1.88	0.98 ± 1.55	0.067
	Female (211)	BMI	21.73 ± 6.65	20.36 ± 5.08	0.277
		BMI Z-score	0.38 ± 1.62	0.22 ± 1.59	0.621

**Abbreviations:** BMI, body mass index;

**Table 4:** Prevalence of Subclinical hypothyroidism in each BMI category

		Underweight	Healthy weight	Overweight	Obese	P
All ages	All subjects	9.9%	13.8%	17.2%	20.5%	0.032*
	Male subjects	10.1%	14.3%	11.9%	24.3%	0.037*
	Female subjects	9.7%	13.6%	20.3%	16.8%	0.297
2-9 year olds	All subjects	9.6%	12.1%	23.9%	24.8%	0.005*
	Male subjects	11.1%	15.9%	0.0%	30.0%	0.065
	Female subjects	7.9%	10.3%	28.2%	21.3%	0.011*
10-18 year olds	All subjects	10.3%	15.9%	12.9%	17.1%	0.624
	Male subjects	8.8%	12.9%	14.3%	21.1%	0.345
	Female subjects	12.5%	18.0%	11.4%	11.5%	0.642

**Abbreviations:** BMI, body mass index;

**Table 5:** Distribution of euthyroid and subclinical hypothyroid children based on their BMI category

		All ages			2-9 year olds			10-18 year olds		
		Euthyroid	Subclinical Hypothyroid	P	Euthyroid	Subclinical Hypothyroid	P	Euthyroid	Subclinical Hypothyroid	P
All subjects	Underweight	17.7%	10.7%	0.032*	20.8%	11.8%	0.005	14.5%	9.5%	0.624
	Healthy weight	44.2%	38.9%		48.5%	35.3%		39.9%	42.9%	
	Overweight	13.4%	15.3%		9.7%	16.2%		17.0%	14.3%	
	Obese	24.8%	35.1%		21.1%	36.8%		28.5%	33.3%	
Male subjects	Underweight	23.2%	13.6%	0.037*	31.3%	18.5%	0.065	17.4%	9.4%	0.345
	Healthy weight	37.3%	32.2%		41.4%	37.0%		34.3%	28.1%	
	Overweight	12.1%	8.5%		5.5%	0.0%		16.9%	15.6%	
	Obese	27.5%	45.8%		21.9%	44.4%		31.5%	46.9%	
Female subjects	Underweight	13.6%	8.3%	0.297	15.0%	7.3%	0.011*	11.7%	9.7%	0.642
	Healthy weight	49.4%	44.4%		52.4%	34.1%		45.6%	58.1%	
	Overweight	14.3%	20.8%		12.0%	26.8%		17.2%	12.9%	
	Obese	22.8%	26.4%		20.6%	31.7%		25.6%	19.4%	

**Abbreviations:** BMI, body mass index

## Results

Table 1 shows anthropometric and laboratory values of subjects in different BMI and thyroid categories. Subjects had a statistical difference in their age and gender. Therefore, these parameters had to be adjusted when calculating the correlation between TSH and BMI Z-score. Median and interquartile range (IQR) of time of day at which the subjects had their blood samples taken was 13:00 (IQR: 9: 40-16:25) for euthyroid subjects and 11:25 (IQR: 8: 35-15:12) for subclinical hypothyroid subjects. No significant difference could be found between the two based on Mann-Whitney U test (*p* = 0.070).

Table 2 demonstrates mean thyroid parameters in the study subjects based on their respective BMI group. A statistically significant increase in mean TSH levels in higher BMI groups is demonstrated. It’s important to note that when comparing mean TSH levels in the age and gender subgroups, the difference is statistically significant in the overall male group, overall 2-9 year old age group and the male 2-9 year old age group.

Table 3 shows mean BMI parameters in euthyroid and subclinical hypothyroid children. A statistically significant difference is found in mean BMI and BMI Z-score when comparing euthyroid and subclinical hypothyroid children. The table also illustrates that when comparing mean BMI Z-score in the age and gender subgroups, the difference between thyroid groups is statistically significant in the overall female group, overall 2-9 year old age group and the female 2-9 year old age group.

When TSH is set as a dependent value (effect of BMI Z-score on TSH levels) for correlation of TSH levels and BMI Z-score based on linear regression, TSH level is increased approximately 0.126 mIU/L with every 1 score increase in BMI Z-score with a correlation coefficient of 0.125 adjusted for age and gender (*p* = 0.001). However, When BMI Z-score is set as a dependent value (effect of TSH level on BMI Z-score), BMI Z-score is increased approximately 0.113 with every 1 mIU/L increase in TSH levels with a correlation coefficient of 0.243 (p = 0.001). Adjusted linear correlation of TSH and BMI Z-score was also statistically significant in the 2-9 year old age group (TSH dependent value, r = 0.185, β = 0.164, *p* = 0.000) and the male group (TSH dependent value, r = 0.176, β = 0.157, p = 0.001) while this was not true in the 10-18 year old age group (*p* = 0.132) and the female group (*p* = 0.078).

Based on Table 4, the prevalence of subclinical hypothyroidism is increased in higher BMI groups. 9.9, 13.8, 17.2 and 20.5% of underweight, healthy weight, overweight and obese had subclinical hypothyroidism. On the other hand, based on Table 5, subclinical hypothyroid subjects have a higher prevalence of overweight or obesity than euthyroid subjects. (Table 4 and Table 5).

Based on logistic regression adjusted for age and gender, obese and overweight participants had 1.649 (CI95%: 1.126–2.413, *p* = 0.010) times higher odds of subclinical hypothyroidism than those who were not obese or overweight. On the other hand, Subclinical hypothyroid participants had 1.650 (CI95%: 1.128–2.413, p = 0.010) times higher odds of overweight or obesity than those who were euthyroid.

**Fig. 1:**
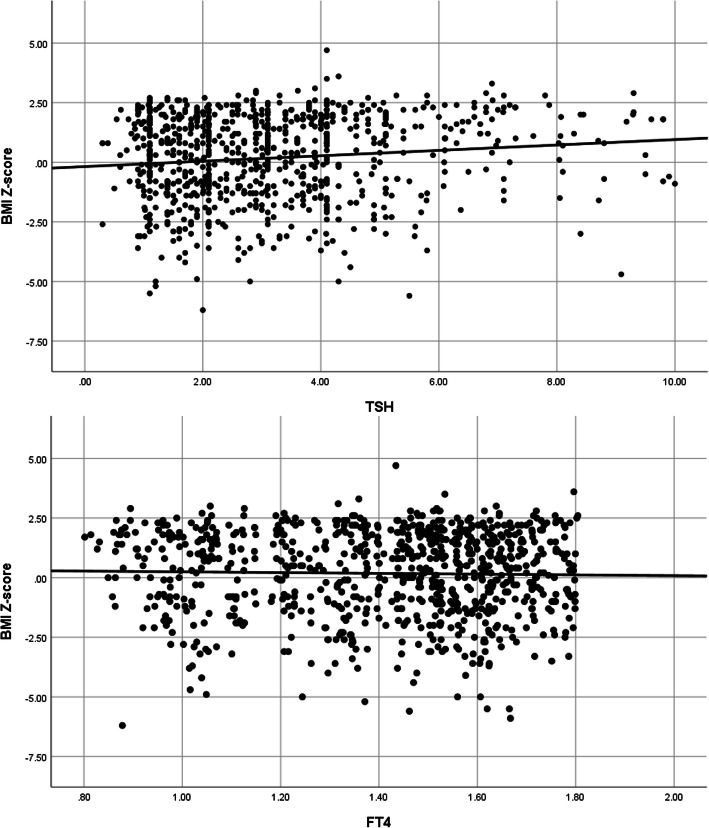
Linear correlation of BMI Z-score with TSH and FT4 levels in all 850 participants of this study

Figure 1 shows the scatter plot of subjects in this study based on their BMI Z-score, TSH and free T4 levels. (Fig. 1) A significant linear correlation between seum TSH levels and BMI Z-score is demonstrated.

## Discussion

This study reveals that a linear correlation exists between serum TSH levels and BMI Z-score in children without overt hypothyroidism especially in those under the age of 10. The study also reveals the prevalence of subclinical hypothyroidism in southern Iranian children based on their BMI status.

Prevalence of subclinical hypothyroidism in adults is reported to be in a range of 1 to 12.5% [3, 28]. In children, little information is available on the prevalence of SH. In various studies, the prevalence rate ranged between 1.7 to 9.5% [28]. In the study by Jin HY, the prevalence rate was 12.8% in healthy children and 24.3% in obese children [29] and in our study, it was 13.8% in healthy children and 20.5% in obese children. The reason for this amount of variation in prevalence rate is probably due to either different selection of cut-offs for a normal or abnormal range of serum TSH levels or once vs more than once measurements of serum TSH levels in the study participants.

In most studies, SH has a higher prevalence in overweight and obese children [9, 11, 29–34].

According to Marras V et al., of the 468 obese participants, 109 had abnormal thyroid hormone concentrations. After 6 months of lifestyle intervention in 43 participants, thyroid hormones normalized in 27 of the patients with decreased BMI Z-score [9]. Reinehr T et al. found that although obesity and higher TSH levels are correlated, lipid serum components had no effect on serum TSH levels. Weight loss in 49 of the 246 obese participants led to a significant reduction of TSH [11].

Jin HY, however, found that besides TSH levels being positively correlated with BMI Z-score, the concentrations of total cholesterol and triglyceride were also positively correlated with the TSH concentrations following adjustment for age and BMI Z-score [29].

In the study by Stitche H, TSH and T3 levels are significantly increased in obese children. Obesity had no effect on serum T4, urinary iodine excretion, and Anti-TPO Ab, however [30].

In a study by Ghergherehchi R et al. on 323 children in Iran, 14.7% of obese participants had subclinical hypothyroidism, which is lower than our results, while 6.8% of normal subjects had SH. BMI Z-score was positively correlated with TSH levels [31].

According to a study by Bhowmick SK et al., 11.7% of obese participants had SH compared to 0.7% of healthy weight children. Mean TSH levels in non-positive Anti TPO Ab obese participants was 5.33 mIU/L. [33].

In another study by Dekelbab BH, 10.8% of 185 obese participants with negative Anti TPO Ab had TSH levels higher than 4 mIU/L. Mean TSH in the subjects was 7.51 mUI/L. [34].

According to our findings, there is a significant statistical difference between BMI Z-score in euthyroid vs subclinical hypothyroid children. The average BMI Z-score for euthyroid children is 0.102 ± 1.8334 and for subclinical hypothyroid children is 0.595 ± 1.8076 (*P* = 0.005).

In other words, with an increase in BMI Z-score, TSH level is increased, and on the other hand, as the TSH levels increase, the prevalence of higher BMI Z score also increases. Which one is the cause and which is the causality? The correct answer to this question cannot be given. However, while in our study the correlation coefficient is stronger when TSH is set as an independent value and BMI Z-score as a dependent, in many studies weight loss leads to a lower TSH level [10–15] and in a study by Knudsen N et al., a positive correlation between weight gain over 5 years and a progressive increase in TSH was noted [35]. Based on this fact, Peeters RP believes that subclinical hypothyroidism is an unlikely cause of obesity [36]. Another hypothesis based on this assumption is that because elevated serum TSH levels are reversible after weight loss, this may not be ‘true’ subclinical hypothyroidism and may possibly be a result of adaptive responses to thyrotropic feedback control i.e. obesity leading to increase in serum leptin-mediated Pro TRH [17–19] and increase in resting energy expenditure [10, 16] which in turn, leads to a higher serum TSH level. However, what we don’t know at the time of this study is that do any of these ‘reversible’ subclinical hypothyroid patients eventually lead to overt hypothyroidism in the long term? To answer this question, a long term prospective study is needed on subjects demonstrating decrease in TSH levels after weight loss.

Meanwhile, Manji N et al. found no correlation between TSH levels and BMI Z-score in euthyroid participants [37].

### Study limitations

The most important limitation of this study is that while it illustrates a positive link between serum TSH levels and BMI Z-score, it does not reveal the causality of this link. Is obesity causing higher levels of serum TSH, or is progression of subclinical hypothyroidism causing weight gain? Based on our study, the correlation coefficient is higher when BMI Z-score is set as the dependent value. This may mean that it is subclinical hypothyroidism that is causing obesity, not the other way around. However, this is not definite and decrease in serum TSH levels during weight loss as seen in other studies may counter that hypothesis. More research on the cause and causality of obesity and subclinical hypothyroidism should be performed. Another limitation is that while Anti-TPO Ab levels are not required for a subclinical hypothyroidism diagnosis, they are recommended for considering therapeutic treatment of the disease and they would have certainly helped in a better analysis of the subjects in this study. This study also did not record subject puberty status, which may affect serum TSH levels. Regarding the time span at which the blood specimen were collected, it should be noted that despite the diurnal variation in TSH levels, this variation will not influence the diagnostic interpretation of results since reference intervals for TSH are established with the variation between 8 AM and 6 PM in mind [38]. We found no significant difference in proportion of subclinical hypothyroid patients in morning (18.3% of *n* = 229) vs evening (11.3% of *n* = 124) shifts (*p* = 0.083) and the time of day sampling between euthyroid and subclinical hypothyroid subjects (change of shift at 2:30 PM).

## Conclusion

Prevalence of subclinical hypothyroidism is higher in overweight and obese children and increase in BMI Z-score and serum TSH levels are closely linked. However, because weight loss results in normalization of serum TSH levels, it can be assumed that elevated TSH levels in these subjects are due to adaptive responses of thyrotropic feedback control to obesity rather than “true” hypothyroidism. Furthermore, is the treatment of subclinical hypothyroidism in children also effective for weight loss? That is unclear and more extensive studies are needed to evaluate the eventual outcome of SH in children. Finally, based on results of this study, a thyroid profile test should be considered in approach to obesity in children. If subclinical hypothyroidism is found in obese children, a weight loss plan may lead to a decrease in serum TSH levels and a drug therapy may not be needed.

## Data Availability

The datasets used during the current study are not available due to confidentiality policy set by the clinic.

## Abbreviations

BMI: body mass index
TSH: thyroid stimulating hormone
FT4: free T4
CI: confidence interval
CDC: center for disease control and prevention
SH: subclinical hypothyroidism
IQR: interquartile range
